# Molecular investigation on diversity of the land snail genus *Aegista* (Gastropoda, Camaenidae) in South Korea

**DOI:** 10.3897/BDJ.11.e96800

**Published:** 2023-01-31

**Authors:** Kazuki Kimura, Satoshi Chiba, Jae-Hong Pak

**Affiliations:** 1 Center for Northeast Asian Studies, Tohoku University, Sendai, Japan Center for Northeast Asian Studies, Tohoku University Sendai Japan; 2 Department of Biology, Kyungpook National University, Taegu, South Korea Department of Biology, Kyungpook National University Taegu South Korea

**Keywords:** *Aegista*, cryptic species, land snails, molecular phylogeny, the Korean Peninsula

## Abstract

*Aegista* Albers, 1850 is a large genus of the land snail family Camaenidae Pilsbry, 1895 and distributed in south, southeast and east Asian countries (from India and Nepal to Korea and Japan). Fourteen species and subspecies of *Aegista* are known from South Korea. They were described, based only on shell morphology during 1887–1943 and our knowledge on diversity of Korean *Aegista* has seldom been updated since then. In this study, we provide the report on the first molecular investigation of diversity of *Aegista* in South Korea, which unmasked some of overlooked diversity of this group.

## Introduction

**Figure 1.**
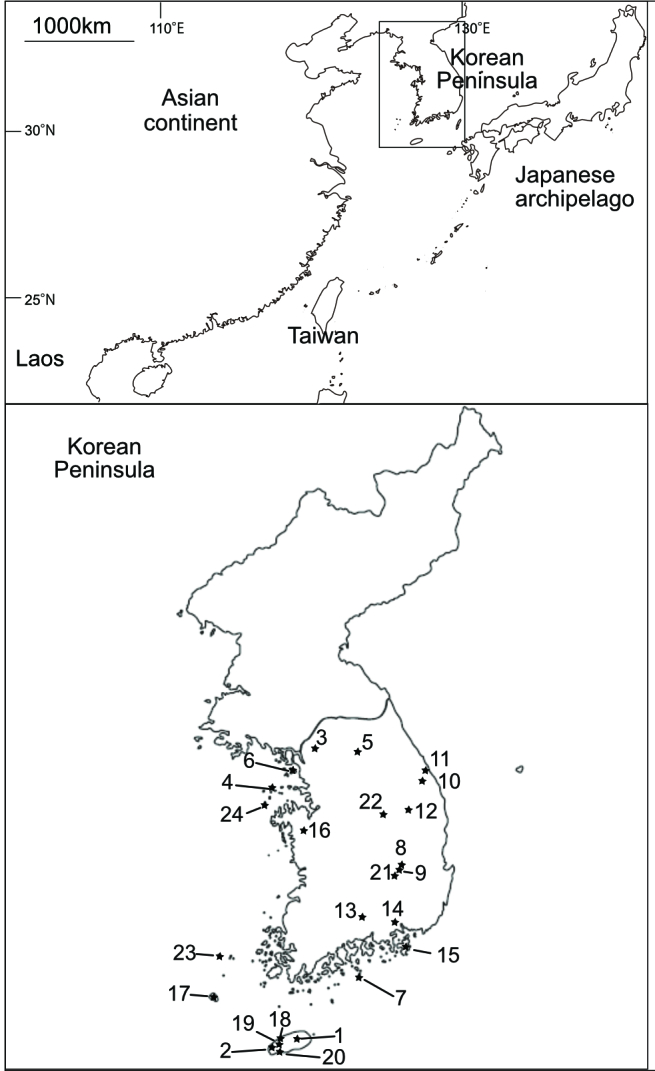
Map showing the sampling localities. The number corresponds to the locality No. in Table 1. Our analyses also included the sequences of the specimens from Japan, Taiwan and Laos.

The Korean Peninsula is located at the edge of the eastern part of the Asian continent (Fig. 1). It has had intermittent land-bridge connections with mainland China and its nearby large archipelago (i.e. the Japanese archipelago) through multiple glacial cycles (Kim and Kennett 1998, Kong et al. 2006). Moreover, the Peninsula has never been covered by ice sheets during the Quaternary, which makes it possible for terrestrial organisms to inhabit the Peninsula for a long period (Chung et al. 2017). In East Asia, for example, the Korean Peninsula is the only region where an ancient divided member of the salamander family Plethodontidae Gray, 1850 (Amphibia), whose centre of diversity is North and Middle America, is found (Min et al. 2005). The ancestral group of the Asian plethodontid salamander is estimated to have migrated from the North American continent around 45 Mya and Asian plethodontid populations have probably been extirpated, except in the climatologically stable region, the Korean Peninsula (Shen et al. 2015). These complex geographical characteristics make this region attractive for biodiversity and phylogeographic studies.

*Aegista* Albers, 1850 is a large genus of the land snail family Camaenidae Pilsbry, 1895 and distributed in south, southeast and east Asian countries (from India and Nepal to Korea and Japan) (Hirano et al. 2015, Nurinsiyah et al. 2019). Although some researchers treat *Landouria* Godwin-Austen, 1918 as a distinct genus (Köhler et al. 2018, Nahok et al. 2021), as suggested by Hirano et al. (2014), we treated members of the genus as *Aegista* in this study because the monophyly of *Landouria* is still unclear. Fourteen species and subspecies of *Aegista* are known from South Korea (Kuroda 1958). They were described during 1887–1943 and, unfortunately, our knowledge on diversity of Korean *Aegista* has seldom been updated since then. The classification of these Korean *Aegista* is based only on shell morphology. However, recent molecular phylogenetic studies of land snails have revealed cryptic species within a single morphospecies and demonstrated the unreliability of shell morphology for species identification (e.g. Chiba and Davison (2008), Nantarat et al. (2014)). Hirano et al. (2014) examined a lot of species of *Aegista* (n >70) from almost all regions of Japan in addition to its surrounding regions such as Taiwan and China and found that multiple phylogenetic clades inhabit the Japanese archipelago, which has a profound biogeographic connection with the Korean Peninsula. Whether the Korean *Aegista* species include cryptic species and distribution pattern of the phylogenetic lineages in the Korean Peninsula remain to be studied. In the present study, we aim to address this question.

## Material and methods

### Samples

**Figure 2.**
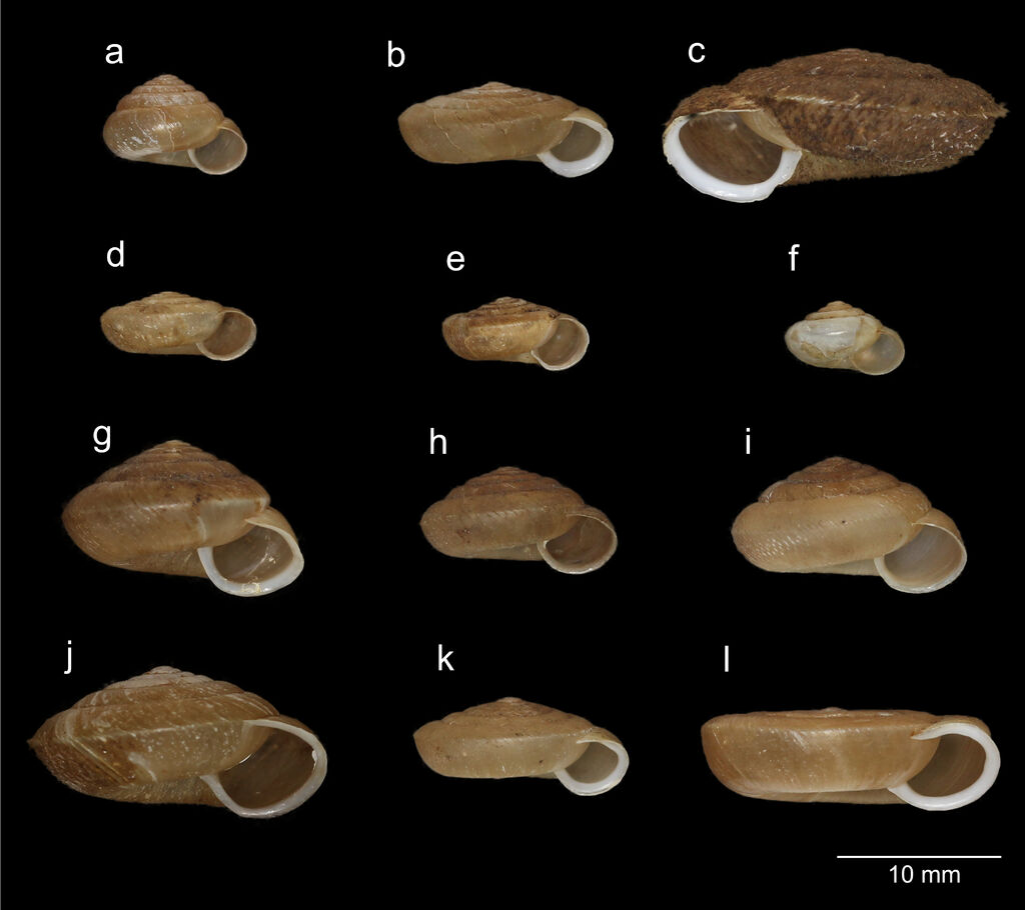
Shells of the Korean *Aegista* species used in this study. **a** *A.chejuensis*; **b** *A.chosenica*; **c** *A.diversa*; **d** *A.gottscheigottschei*; **e** *A.gottscheikyobuntonis*; **f** *A.ottoi*; **g** *A.proxima*; **h** *A.pyramidatapyramidata*; **i** *A.pyramidatahebes*; **j** *A.quelpartensis*; **k** *A.tenuissimatenuissima*; **l** *A.tenuissimaomorii*.

Individuals of 12 *Aegista* species and subspecies (Fig. 2) were collected in South Korea from 2018 to 2021 (Table 1). In total, 42 individuals were used in this study and six of them were not identified to the species-level because they were still small juveniles. A small portion of the foot muscle from each snail individual was stored in 99.5% ethanol for DNA extraction. For some species, DNA sampling with body swabbing (Morinha et al. 2014) was used in order to avoid unwanted effects of sample collecting on snail populations. The examined *Aegista* specimens are preserved in Tohoku University Museum (TUMC) or the authors’ collection (Voucher No. in Table 1).

### Phylogenetic analyses

Total DNA was isolated from each foot piece and swabbed subsance using the DNeasy Blood & Tissue Kit or QIAamp DNA Micro Kit (Qiagen), according to the manufacturer’s instructions. Fragments of mitochondrial (cytochrome oxidase subunit I [COI] gene) and nuclear (internal transcribed spacer [ITS] 1-2 regions and 5.8S rRNA gene) DNA markers were amplified and sequenced. The PCR conditions and primer sets were decided following Hirano et al. (2014). The PCR products were purified using Exo-SAP-IT (Amersham Biosciences, Little Chalfont, Buckinghamshire, UK). Sequencing was performed using a BigDye™ Terminator Cycle Sequencing Ready Reaction Kit (Applied Biosystems, Foster City, CA, USA) and electrophoresed using an ABI 3130xl sequencer (Applied Biosystems, Carlsbad, CA, USA). The resulting COI and ITS sequences have been deposited in the DDBJ/EMBL/GenBank database (Table 1). In addition to these new sequence data for Korean *Aegista*, already existing datasets of the genus and its closely related genus *Euhadra* Pilsbry, 1890 were obtained from GenBank in order to conduct the phylogenetic analyses (Table 1).

The mitochondrial and nuclear sequences were aligned with MUSCLE v.3.8 (Edgar 2004). In order to eliminate any uncertainty in the ITS alignments, trimAl (v. 1.2) with automated option (Capella-Gutiérrez et al. 2009) was used to exclude ambiguous alignment regions. The phylogenetic trees were obtained for a concatenated dataset (533 sites for COI and 1,147 sites for ITS) using Maximum Likelihood (ML) and Bayesian Inference (BI) methods. Prior to the ML and BI analyses, ModelFinder (Kalyaanamoorthy et al. 2017) was used to select the optimal partition scheme for codon position and the appropriate models for sequence evolution. As a result, the following models were selected: TIM2+F+G4 model for codon 1 of COI, TIM+F+R4 for codon 2 of COI, GTR+F+ASC+G4 for codon 3 of COI, TVM+F+R3 for ITS in the ML analysis; GTR+G for codons 1, 2 and 3 of COI and ITS in the BI analysis. ML analysis was performed with IQ-TREE (v. 1.6.8) using the options of edge-unlinked branch lengths between partitions and a perturbation strength of 1.0 (Nguyen et al. 2014, Chernomor et al. 2016). For the ML analyses, we assessed nodal support by performing ultrafast bootstrap analyses with 10,000 replications in IQ-TREE (Hoang et al. 2018). BI analysis was performed using MrBayes (v. 3.2.7) with two simultaneous runs (Ronquist and Huelsenbeck 2003). Each run consisted of four simultaneous chains for two million generations and sampling of trees every 100 generations. We discarded the first 2,001 trees as burn-in after examining the convergence of runs and their effective sample sizes (ESSs) using Tracer (v. 1.6) and used the remaining samples to estimate tree topology, branch length and substitution parameters.

### Species delimitation analyses

To validate the Korean species of *Aegista*, three species delimitation analyses were conducted. For topology-based approach, ML Poisson Tree Process model (mlPTP; Zhang et al. (2013)) and the Bayesian Poisson Tree Process model (bPTP; Zhang et al. (2013)) were used and the topology of the obtained ML tree was applied for both models. For the bPTP model, we set default parameters (number of MCMC generation: 100,000; burn-in: 10%). Both analyses were run using web servers (http://species.h-its.org/ptp/). For distance-based approach, Assemble Species by Automatic Partitioning (ASAP) analysis (Puillandre et al. 2020) was performed using the COI dataset. This analysis was run using Kimura (K80) distances with the default setting at the ASAP website (https://bioinfo.mnhn.fr/abi/public/asap/asapweb.html). The partitions with the first and second-best asap-scores were considered according to Puillandre et al. (2020).

## Results

The results of the phylogenetic analyses for the concatenated sequences using ML and BI methods were mostly consistent. Only well-supported clades (the posterior probabilities ≥ 0.95 or ultrafast bootstrap support values ≥ 90%) are considered hereafter.

**Figure 3.**
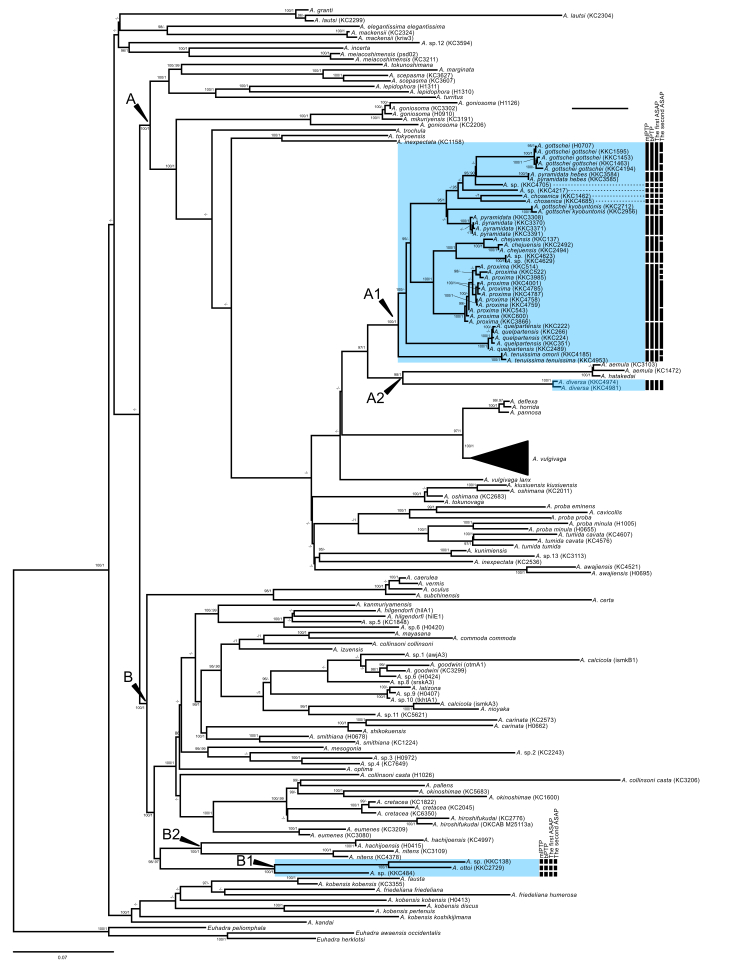
ML tree of the *Aegista* species, based on the concatenated data (1680 bp) of the COI and ITS DNA markers. Each OTU label represents a species name. Numbers at nodes represent Bayesian posterior probabilities (right) and Maximum Likelihood ultrafast bootstrap support values (left). Only Bayesian posterior probabilities ≥ 0.95 and bootstrap support values ≥ 90% are shown. Scale bar indicates 0.07 substitutions per site. Arrow heads indicate clades and subclades. Blue shadows mark the Korean *Aegista* species. Black bars represent putative species suggested by the species delimitation analyses.

The 12 *Aegista* species in South Korea were separated into two major clades (Fig. 3). Subclade A1 was composed of at least nine species of Korean *Aegista* and subclade A2 was composed of a Korean and two Japanese species. Subclade B2 included the remaining Korean species and showed a sister relationship with subclade B1 composed of two Japanese species. The monophyly of *A.chosenica* was supported only in the BI tree. Neither the pairs of the subspecies of *A.gottschei* (i.e. *A.gottscheigottschei* and *A.gottscheikyobuntonis*) nor *A.pyramidata* (i.e. *A.pyramidatapyramidata* and *A.pyramidatahebes*) showed any sister relationship.

For the Korean species in subclade A1, A2, and B2 the mlPTP, bPTP and ASAP analyses provided the same pattern of species delimitation (Fig. 3) and delineated 17 species (asap-score for the ASAP: 5.00), while the ASAP also partitioned 26 species with the second-best asap-score (6.50). For all analyses, the number of the species delineated was higher than the number of the Korean *Aegista* species recognised. None of the six small individuals that we could not identify was treated as a species with the identified snails in the species delimitation analyses.

## Discussion

Our phylogenetic analyses, using the COI and ITS markers, revealed that multiple phylogenetic lineages of *Aegista* inhabit the Korean Peninsula and they are included in clade A or B. Hirano et al. (2014) showed that the majority of the *Aegista* species in the mainland of Japan are included in the two clades. Our result provides an example showing a close relationship between terrestrial biotas of Japan and South Korea. In both clades A and B, the lineages of Korean *Aegista* are not ancestral, which may suggest dispersal events of their progenitors from the Japanese archipelago to the Korean Peninsula. To test this hypothesis, further studies are needed to examine when *Aegista* has diversified and compare it with the geohistorical background of the Asian continent and the Japanese archipelago. Discovery of fossils of *Aegista* or big data arising from NGS technologies will help to address the history of diversification of this genus. Of the 14 *Aegista* species and subspecies known from South Korea, phylogenetic positions of *A.pumilio* (Pilsbry & Hirase, 1909) and *A.gottscheifusanica* (Pilsbry, 1926) are unclear. Although the unidentified small individuals may include these two taxa, further examination using specimens of them is needed to clarify their phylogenetic relationship with the other Korean *Aegista*.

The species delimitation analyses suggested the presence of cryptic species in *A.chosenica* (Fig. 3). In addition, each of *A.gottscheikyobuntonis* and *A.pyramidatahebes* would need to be treated as a distinct species. Kuroda (1958) proposed the scientific name *A.pyramidatahebesioides* Kuroda, 1958 for the latter because *A.hebes* (Pilsbry & Hirase, 1905) is known from Taiwan. Although Kuroda’s proposal based on the comparison between species and subspecies and thus was regarded as unnecessary, it would need to be reconsidered. The analyses also suggested that several unrecognised species of *Aegista* inhabit South Korea. It seems that at least three species have been overlooked even if the unidentified individuals include *A.pumilio* and *A.gottscheifusanica*.

Previous studies exhibited that *Aegista* shows a striking divergence in shell morphology and provides an example of parallel evolution of morphological traits (Hirano et al. 2014, Hirano et al. 2015). Our results also found examples of the independent evolution of shell flatness and hair-like ornamentation of the shell. For example, *A.tenuissimaomorii* has a flat shell, while *A.chejuensis* has a globular shell.

## Conclusions

In this study, we provide the report on first molecular investigation of diversity of *Aegista* in South Korea, which unmasked some of overlooked diversity of this group. To fully understand diversity of Korean *Aegista*, further studies using additional specimens including *A.pumilio* and *A.gottscheifusanica*, and an additional sampling effort, especially in the central region of the Korean Peninsula, are required.

## Figures and Tables

**Table 1.** List of the species included in the molecular phylogenetic analyses. Asterisks indicate the sequences newly obtained in this study.

Species names	Locality	Locality No	Voucher #	GenBank (COI)	GenBank (ITS)
*A.aemula* (Gude, 1900)	Kusama, Niimi		KC3103	AB852664	AB852931
*A.aemula*	Tojyo, Shobara		KC1472	AB852665	AB852932
*A.awajiensis* (Gude, 1900)	Yashima, Takamatsu		H0695	AB852625	AB852892
*A.awajiensis*	Naka		KC4521	AB852624	AB852891
*A.caelurea* Kuroda & Habe, 1960	Ishigaki, Okinawa		Aeg02	AB852626	AB852893
*A.calcicola* (Kuroda, 1989)	Ishimaki, Toyohasi		ismkB1	AB852712	AB852982
*A.calcicola*	Inasa, Hamamatsu		ismkA3	AB852713	AB852983
*A.carinata* (Gude, 1901)	Monobe, Kami		KC2573	AB852714	AB852984
*A.carinata*	Naka		H0662	AB852715	AB852985
*A.cavicollis* (Pilsbry, 1900)	Sakyo, Kyoto		KC7121	AB852661	AB852928
*A.certus* (Zilch, 1938)	Mt. Nanren, Henchung		KC2953	AB852704	AB852974
*A.chejuensis* (Pilsbry & Hirase, 1908)	Pongedong, Cheju	1	KKC137	LC742391*	LC742435*
*A.chejuensis*	Hangyeong, Cheju	2	KKC2492	LC742407*	LC742451*
*A.chejuensis*	Hangyeong, Cheju	2	TUMC112741	LC742408*	LC742452*
*A.chosenica* (Pilsbry, 1927)	Mt. Soyo, Kyongido	3	KKC1462	LC742403*	LC742447*
*A.chosenica*	Tokchokk Island, Incheon	4	KKC4685	LC742426*	LC742470*
*A.collinsonicasta* (Pilsbry, 1901)	Kamikoshiki, Kagoshima		H1026	AB852716	AB852986
*A.collinsonicasta*	Mt. Inao, Minamiosumi		KC3206	AB852717	AB852987
*A.collinsonicollinsoni* (Adams, 1868)	Kushimoto		tsnmA2	AB852718	AB852988
*A.commodacommoda* (Adams, 1868)	Yasuda, Shodoshima		H0417	AB852719	AB852989
*A.commodanioyaka* (Pilsbry & Hirase, 1904)	Oya, Shizuoka		H0398	AB852720	AB852990
*A.cretacea* Gude, 1900	Yoshii, Ibara		KC1822	AB852721	AB852991
*A.cretacea*	Tojyo, Shobara		KC2045	AB852722	AB852992
*A.cretacea*	Bessho, Izumo		KC6350	AB852723	AB852993
*A.cretacea*	Mt. Hakugaku, Kure		KC2776	AB852724	AB852994
*A.deflexa* Pilsbry, 1902	Tobishima Island, Sakata		KC2990	AB852666	AB852933
*A.diversa* Kuroda & Miyanaga, 1936	Namsan, Chunchon	5	KKC4974	LC742433*	LC742477*
*A.diversa*	Namsan, Chunchon	5	TUMC112742	LC742434*	LC742478*
*A.elegantissimaelegantissima* (Pfeiffer, 1849)	Mt. Hedo, Kunigami		KC9200	AB852667	AB852935
*A.eumenes* (Westerlund, 1883)	Amidadera, Shimonoseki		KC3209	AB852725	AB852995
*A.eumenes*	Goya, Tagawa		KC3080	AB852726	AB852996
*A.fausta* Kuroda & Habe, 1951	Mt. Toma, Hatsukaichi		KC7539	AB852627	AB852894
*A.friedelianafriedeliana* (von Martens, 1864)	Goya, Tagawa		KC3072	AB852628	AB852895
*A.friedelianahumerosa* (Pilsbry & Hirase, 1904)	Kamikoshiki, Kagoshima		H1024	AB852629	AB852896
*A.goodwini* (Smith, 1876)	Akasaka, Ogaki		otmA1	AB852727	AB852997
*A.goodwini*	Sakyo, Kyoto		KC3299	AB852728	AB852998
*A.gottschei* (Möllendorff, 1887)	Incheon		H0707	AB852630	AB852897
*A.gottscheigottschei*	Mt. Soyo, Kyongido	3	TUMC112743	LC742402*	LC742446*
*A.gottscheigottschei*	Mt. Soyo, Kyongido	3	KKC1463	LC742404*	LC742448*
*A.gottscheigottschei*	Chumk, Incheon	6	KKC1595	LC742405*	LC742449*
*A.gottscheigottschei*	Mt. Soyo, Kyongido	3	KKC4194	LC742422*	LC742466*
*A.gottscheikyobuntonis* Kuroda & Miyanaga, 1943	Kobun Island, Chollanamdo	7	KKC2712	LC742409*	LC742453*
*A.gottscheikyobuntonis*	Kobun Island, Chollanamdo	7	KKC2956	LC742411*	LC742455*
*A.granti* (Pfeiffer, 1865)	Wulai, Taipei		KC2965	AB852631	AB852898
*A.hachijoensis* (Pilsbry, 1902)	Kurio, Yakushima		KC4997	AB852729	AB852999
*A.hachijoensis*	Satamisaki, Minamiosumi		H0415	AB852730	AB853000
*A.hatakedai* Kuroda & Habe, 1951	Kusama, Niimi		KC7286	AB852668	AB852936
*A.hilgendorfi* (Kobelt, 1879)	Akasaka, Ogaki		hilA1	AB852731	AB853001
*A.hilgendorfi*	Ibuki, Maibara		hilE1	AB852732	AB853002
*A.horrida* (Pilsbry, 1900)	Yunohama, Tsuruoka		H0429	AB852669	AB852937
*A.incertus* (Pfeiffer, 1866)	Wulai, Taipei		KC2968	AB852705	AB852975
*A.inexpectata* Kuroda & Minato, 1977	Ishimaki, Toyohashi		KC2536	AB852670	AB852938
*A.inexpectata*	Nippara, Okutama		KC1158	AB852671	AB852939
*A.izuensis* (Pilsbry & Hirase, 1904)	Yugashima, Izu		H0423	AB852733	AB853003
*A.kandai* Azuma, 1970	Yakata Island, Saeki		KC4222	AB852662	AB852929
*A.kanmuriyamensis* Azuma & Azuma, 1982	Sakauchi, Ibigawa		KC2225	AB852632	AB852899
*A.kiusiuensiskiusiuensis* (Pilsbry, 1900)	Onotsu, Kikai		KC4280	AB852672	AB852940
*A.kiusiuensistokunovaga* (Pilsbry & Hirase, 1905)	Akirigami, Kagoshima		KC4381	AB852673	AB852941
*A.kobensisdiscus* (Pilsbry & Hirase, 1904)	Tosayama, Kochi		KC1245	AB852633	AB852900
*A.kobensiskobensis* (Schmacker & O. Boettger, 1890)	Ashiu, Nantan		KC3355	AB852634	AB852901
*A.kobensiskobensis*	Yasuda, Shodoshima		H0413	AB852635	AB852902
*A.kobensiskoshikijimana* (Pilsbry & Hirase, 1904)	Kamikoshiki, Kagoshima		H1138	AB852636	AB852903
*A.kobensispertenuis* (Pilsbry & Hirase, 1904)	Hiburi Island, Ehime		KC1618	AB852637	AB852904
*A.kunimiensis* Azuma & Azuma, 1982	Kunimi, Shizukuishi		KC2242	AB852638	AB852905
*A.latizona* (Kuroda & Habe, 1949)	Toubara, Kishiwada		krobA1	AB852734	AB853004
*A.lautsi* (Schmacker & O. Boettger, 1890)	Mt. Nanren, Henchung		KC2304	AB852639	AB852906
*A.lautsi*	Kenting, Henchung		KC2299	AB852640	AB852907
*A.lepidophora* (Gude, 1900)	Mt. Nekumachiji, Ogimi		H1311	AB852674	AB852942
*A.lepidophora*	Mt. Katsuu, Nago		H1310	AB852675	AB852943
*A.mackensii* (Adams & Reeve, 1850)	Ishigaki, Okinawa		KC2324	AB852676	AB852944
*A.mackensii*	Iriomote, Okinawa		kriw3	AB852677	AB852945
*A.marginata* (Pilsbry & Hirase, 1903)	Mt. Hedo, Kunigami		KC2007	AB852678	AB852947
*A.mayasana* (Azuma, 1969)	Mt. Maya, Kobe		H0397	AB852641	AB852908
*A.meiacoshimensis* (Adams & Reeve, 1850)	Ishigaki, Okinawa		psd02	AB852706	AB852976
*A.meiacoshimensis*	Iriomote, Okinawa		KC3211	AB852707	AB852977
*A.mesogonia* (Pilsbry, 1900)	Yasuda, Shodoshima		H0403	AB852735	AB853005
*A.nitens* (Pilsbry & Hirase, 1904)	Mt. Yuwan, Uken		KC3109	AB852736	AB853006
*A.nitens*	Inutabu, Kagoshima		KC4378	AB852737	AB853007
*A.oculus* (Pfeiffer, 1850)	Miyako, Okinawa		H2849	AB852642	AB852909
*A.okinoshimae* (Pilsbry, 1902)	Moshima, Okinoshima		KC5683	AB852738	AB853008
*A.okinoshimae*	Ashizuri, Tosashimizu		KC1600	AB852739	AB853009
*A.optima* (Pilsbry, 1902)	Naka		KC4503	AB852740	AB853010
*A.oshimana* (Pilsbry & Hirase, 1903)	En, Tatsugo		KC2011	AB852679	AB852949
*A.oshimana*	Setouchi, Kagoshima		KC2682	AB852680	AB852950
*A.ottoi* (Pilsbry, 1927)	Kobun Island, Chollanamdo	7	KKC2729	LC742410*	LC742454*
*A.pallens* (Jacobi, 1898)	Oda, Uchiko		KC1818	AB852741	AB853011
*A.pannosa* (Pilsbry, 1902)	Mt. Aoba, Sendai		Aeg05	AB852681	AB852951
*A.probaeminens* (Pilsbry & Hirase, 1904)	Atawa, Mihama		H1234	AB852643	AB852910
*A.probagoniosoma* (Pilsbry & Hirase, 1904)	Inasa, Hamamatsu		H1126	AB852644	AB852911
*A.probagoniosoma*	Toyama, Minamishinano		KC3302	AB852645	AB852912
*A.probagoniosoma*	Kaminago, Shibakawa		KC2206	AB852646	AB852913
*A.probagoniosoma*	Ten’ei		H0910	AB852647	AB852914
*A.probamikuriyensis* (Pilsbry, 1902)	Kuchisakamoto, Shizuoka		KC3191	AB852648	AB852915
*A.probaminula* (Pilsbry, 1900)	Ibuki, Maibara		H1005	AB852649	AB852916
*A.probaminula*	Hatsuchi, Okazaki		H0655	AB852650	AB852917
*A.probaproba* (Adams, 1868)	Kawaradani, Shirahama		KC2268	AB852651	AB852918
*A.proxima* (Pilsbry & Hirase, 1909)	Mt. Palgon, Taegu	8	KKC514	LC742398*	LC742442*
*A.proxima*	Mageundambong, Taegu	9	KKC522	LC742399*	LC742443*
*A.proxima*	Samchok, Kangwondo	10	TUMC112744	LC742400*	LC742444*
*A.proxima*	Samchok, Kangwondo	11	KKC600	LC742401*	LC742445*
*A.proxima*	Yeongju, Kyongsanpukdo	12	KKC3866	LC742418*	LC742462*
*A.proxima*	Sichon, Kyongsannamdo	13	KKC3985	LC742419*	LC742463*
*A.proxima*	Masan, Kyongsannamdo	14	KKC4001	LC742420*	LC742464*
*A.proxima*	Koje Island, Kyongsannamdo	15	KKC4758	LC742428*	LC742472*
*A.proxima*	Koje Island, Kyongsannamdo	15	KKC4759	LC742429*	LC742473*
*A.proxima*	Koje Island, Kyongsannamdo	15	KKC4785	LC742430*	LC742474*
*A.proxima*	Koje Island, Kyongsannamdo	15	KKC4787	LC742431*	LC742475*
*A.pseudopatula* (Möllendorff, 1899)	Gansu		KC9593	AB852652	AB852919
*A.pyramidatahebes* (Pilsbry, 1927)	Seosan, Chunchonnamdo	16	KKC3584	LC742416*	LC742460*
*A.pyramidatahebes*	Seosan, Chunchonnamdo	16	KKC3585	LC742417*	LC742461*
*A.pyramidatapyramidata* (Pilsbry, 1927)	Kageodo, Chollanamdo	17	KKC3308	LC742412*	LC742456*
*A.pyramidatapyramidata*	Kageodo, Chollanamdo	17	TUMC112745	LC742413*	LC742457*
*A.pyramidatapyramidata*	Kageodo, Chollanamdo	17	KKC3371	LC742414*	LC742458*
*A.pyramidatapyramidata*	Kageodo, Chollanamdo	17	KKC3391	LC742415*	LC742459*
*A.quelpartensis* (Pilsbry & Hirase, 1908)	Hallim, Cheju	18	KKC222	LC742393*	LC742437*
*A.quelpartensis*	Hallim, Cheju	18	KKC224	LC742394*	LC742438*
*A.quelpartensis*	Hangyeong, Cheju	19	TUMC112746	LC742395*	LC742439*
*A.quelpartensis*	Andokk, Seogwipo	20	KKC351	LC742396*	LC742440*
*A.quelpartensis*	Hangyeong, Cheju	2	KKC2489	LC742406*	LC742450*
*A.scepasma* (Reeve, 1854)	Iheya, Okinawa		KC3627	AB852682	AB852952
*A.scepasma*	Mt. Nekumachiji, Ogimi		KC3607	AB852683	AB852953
*A.shikokuensis* (Pilsbry & Hirase, 1903)	Tsurugi		H0657	AB852742	AB853012
*A.smithiana* (Pilsbry, 1901)	Haruno, Kochi		H0678	AB852743	AB853013
*A.smithiana*	Tosayamada, Kami		KC1224	AB852744	AB853014
*A.* sp.	Pongedong, Cheju	1	KKC138	LC742392*	LC742436*
*A.* sp.	Talsong, Taegu	21	KKC484	LC742397*	LC742441*
*A.* sp.	Chechon, Chunchonpukdo	22	KKC4217	LC742423*	LC742467*
*A.* sp.	Hon Island, Chollanamdo	23	KKC4623	LC742424*	LC742468*
*A.* sp.	Hon Island, Chollanamdo	23	KKC4629	LC742425*	LC742469*
*A.* sp.	Ul Island, Incheon	24	KKC4705	LC742427*	LC742471*
*A.* sp.1	Totsui, Yura		awjA3	AB852711	AB852981
*A.* sp.10	Takihata, Kawachinagano		tkhtA1	AB852753	AB853024
*A.* sp.11	Kurokawa, Miyata		KC5621	AB852754	AB853025
*A.* sp.12	Muang Kham		KC3594	AB852701	AB852971
*A.* sp.13	Mt. Yudono, Tsuruoka		KC3113	AB852653	AB852920
*A.* sp.2	Kodomari, Nakadomari		KC2243	AB852745	AB853016
*A.* sp.3	Akiu, Sendai		H0972	AB852746	AB853017
*A.* sp.4	Ten’ei		KC7649	AB852747	AB853018
*A.* sp.5	Ogawa, Wakasa		KC1848	AB852748	AB853019
*A.* sp.6	Mt. Myojyo, Itoigawa		H0420	AB852749	AB853020
*A.* sp.7	Dorogawa, Tenkawa		H0424	AB852750	AB853021
*A.* sp.8	Shirasaki, Yura		srskA3	AB852751	AB853022
*A.* sp.9	Mijikano, Katsuragi		H0407	AB852752	AB853023
*A.squarrosatokunoshimana* (Pilsbry & Hirase, 1904)	Inutabu, Kagoshima		KC4945	AB852654	AB852921
*A.subchinensis* (Möllendorff, 1884)	Wulai, Taipei		KC2956	AB852655	AB852922
*A.tenuissimaomorii* Kuroda, 1936	Mt. Soyo, Kyongido	3	KKC4185	LC742462*	LC742465*
*A.tenuissimatenuissima* (Pilsbry & Hirase, 1908)	Namsan, Chunchon	5	TUMC112747	LC742432*	LC742476*
*A.tokyoensis* Sorita, 1980	Bunkyo, Tokyo		H0652	AB852656	AB852923
*A.trochula* (Adams, 1868)	Chikugo, Nagasaki		KC6702	AB852663	AB852930
*A.tumidacavata* (Pilsbry, 1902)	Hikigawa, Shirahama		KC4607	AB852657	AB852924
*A.tumidacavata*	Hongu, Tanabe		KC4576	AB852658	AB852925
*A.tumidatumida* (Gude, 1901)	Takihata, Kawachinagano		H0700	AB852659	AB852926
*A.turritus* (Gude, 1900)	Onna, Okinawa		H0780	AB852710	AB852980
*A.vermis* (Reeve, 1852)	Iriomote, Okinawa		KC2836	AB852660	AB852927
*A.vulgivagalanx* (Pilsbry, 1902)	Suii, Anan		H0666	AB852684	AB852954
*A.vulgivagavulgivaga* (Schmacker & Boettger, 1890)	Mt. Myojyo, Itoigawa		Aeg03	AB852685	AB852955
*A.vulgivagavulgivaga*	Shiramine, Hakusan		H0703	AB852686	AB852956
*A.vulgivagavulgivaga*	Mt. Fujiwara, Inabe		KC1148	AB852687	AB852957
*A.vulgivagavulgivaga*	Taga		KC1949	AB852688	AB852958
*A.vulgivagavulgivaga*	Sakyo, Kyoto		KC2075	AB852689	AB852959
*A.vulgivagavulgivaga*	Nariwa, Takahashi		KC4916	AB852690	AB852960
*A.vulgivagavulgivaga*	Mt. Nachi, Nachikatsuura		KC4596	AB852691	AB852961
*A.vulgivagavulgivaga*	Kumanogawa, Shingu		KC7057	AB852692	AB852962
*A.vulgivagavulgivaga*	Takihata, Kawachinagano		H0698	AB852693	AB852963
*A.vulgivagavulgivaga*	Ishimaki, Toyohashi		Aeg21	AB852694	AB852964
*A.vulgivagavulgivaga*	Inasa, Hamamatsu		KC2105	AB852695	AB852965
*A.vulgivagavulgivaga*	Toyama, Minamishinano		KC3303	AB852696	AB852966
*Euhadraawaensisoccidentalis* Azuma,Tatewaki & Okamura,1987			KC7333	AB852698	AB852968
*Euhadraherklotsi* (von Martens, 1861)			KC3081	AB852699	AB852969
*Euhadrapeliomphala* (Pfeiffer, 1850)			KC1149	AB852700	AB852970
